# Isolation and Structure Characterization of an Antioxidative Glycopeptide from Mycelial Culture Broth of a Medicinal Fungus

**DOI:** 10.3390/ijms151017318

**Published:** 2014-09-29

**Authors:** Jian-Yong Wu, Xia Chen, Ka-Chai Siu

**Affiliations:** Department of Applied Biology & Chemical Technology, State Key Laboratory of Chinese Medicine and Molecular Pharmacology in Shenzhen, The Hong Kong Polytechnic University, Hung Hom, Kowloon, Hong Kong; E-Mails: xiachentiger@gmail.com (X.C.); chai_chai_1987@hotmail.com (K.-C.S.)

**Keywords:** *Cordyceps sinensis*, glycopeptide, structure, antioxidant, cell protection

## Abstract

A novel glycopeptide (Cs-GP1) with an average molecular weight (*M*w) of 6.0 kDa was isolated and purified by column chromatography from the lower *M*w fraction of exopolysaccharide (EPS) produced by a medicinal fungus *Cordyceps sinensis* Cs-HK1. Its carbohydrate moiety was mainly composed of glucose and mannose at 3.2:1.0 mole ratio, indicating an O-linked glycopeptide. The peptide chain contained relatively high mole ratios of aspartic acid, glutamic acid and glycine (3.3–3.5 relative to arginine) but relatively low ratios of tyrosine and histidine. The peptide chain sequence analyzed after trypsin digestion by LC-MS was KNGIFQFGEDCAAGSISHELGGFREFREFLKQAGLE. Cs-GP1 exhibited remarkable antioxidant capacity with a Trolox equivalent antioxidant capacity of 1183.8 μmol/g and a ferric reducing ability of 611.1 μmol Fe(II)/g, and significant protective effect against H_2_O_2_-induced PC12 cell injury at a minimum dose of 10 μg/mL. This is the first report on the structure and bioactivity of an extracellular glycopeptide from the *Cordyceps* species.

## 1. Introduction

Polysaccharide and protein (PSP) complexes from edible and medicinal fungi have attracted increasing interest for their notable bioactivities such as immunomodulation, antitumor and antioxidant [1,2,3]. *Cordyceps* (*Ophiocordyceps*) *sinensis*, generally called the Chinese caterpillar fungus, is a well-known medicinal fungus in traditional Chinese medicine with a wide range of health promoting and therapeutic functions [4,5,6]. Because of the scarcity and high price of natural *C. sinensis* organisms, mycelial fermentation has become a favorable process for mass production of fungal biomass and polysaccharides. Although many previous studies on the bioactive molecules from *C. sinensis* fungi have focused on the polysaccharides either extracted from the fungal mycelia or isolated from the liquid medium, a few have attained proteins and peptides from *C. sinensis* or related species. Wong *et al.* [7] have recently isolated a peptide called Cordymin with a molecular weight of about 10 kDa from *C. militaris* fruit body with strong antifungal activity and antiproliferative activity. This peptide has also been isolated from the mycelia of a *C. sinensis* fungus and shown significant anti-inflammatory and antioxidant effects in animal models [8]. To the best of our knowledge, there is still no reported study on a bioactive peptide produced as an extracellular product by a *Cordyceps* fungus.

Cs-HK1 is a *C. sinensis* fungus isolated from natural fruiting body in our lab and has been applied to mycelial culture and liquid fermentation for production of exopolyssacharide (EPS) [9]. The crude EPS attained by ethanol precipitation from the Cs-HK1 fermentation medium had a protein content of up to 20%–25% (*w*/*w*), which was found to contribute more than the carbohydrate content did to the antioxidant effects of EPS [10]. In a recent study [11], the EPS from the Cs-HK1 fermentation medium was roughly fractionated into different ranges of molecular weight (*M*w) by gradient ethanol precipitation. The lower *M*w fraction attained at a higher ethanol volume ratio (2–5) had a higher protein content and stronger antioxidant activity. In a later study [12], the low *M*w EPS was further fractionated into more pure fractions of PSPs, some of which showed notable antioxidant activities.

The present study was aimed at the purification and structural characterization of an antioxidative glycopeptide from the low *M*w EPS fraction produced by the Cs-HK1 fungus in mycelial liquid culture, and evaluation of its antioxidant property through chemical and cell culture assays.

## 2. Results and Discussion

### 2.1. Isolation and Molecular Profiles of Cs-GP1 from EPS-2

**Figure 1:**
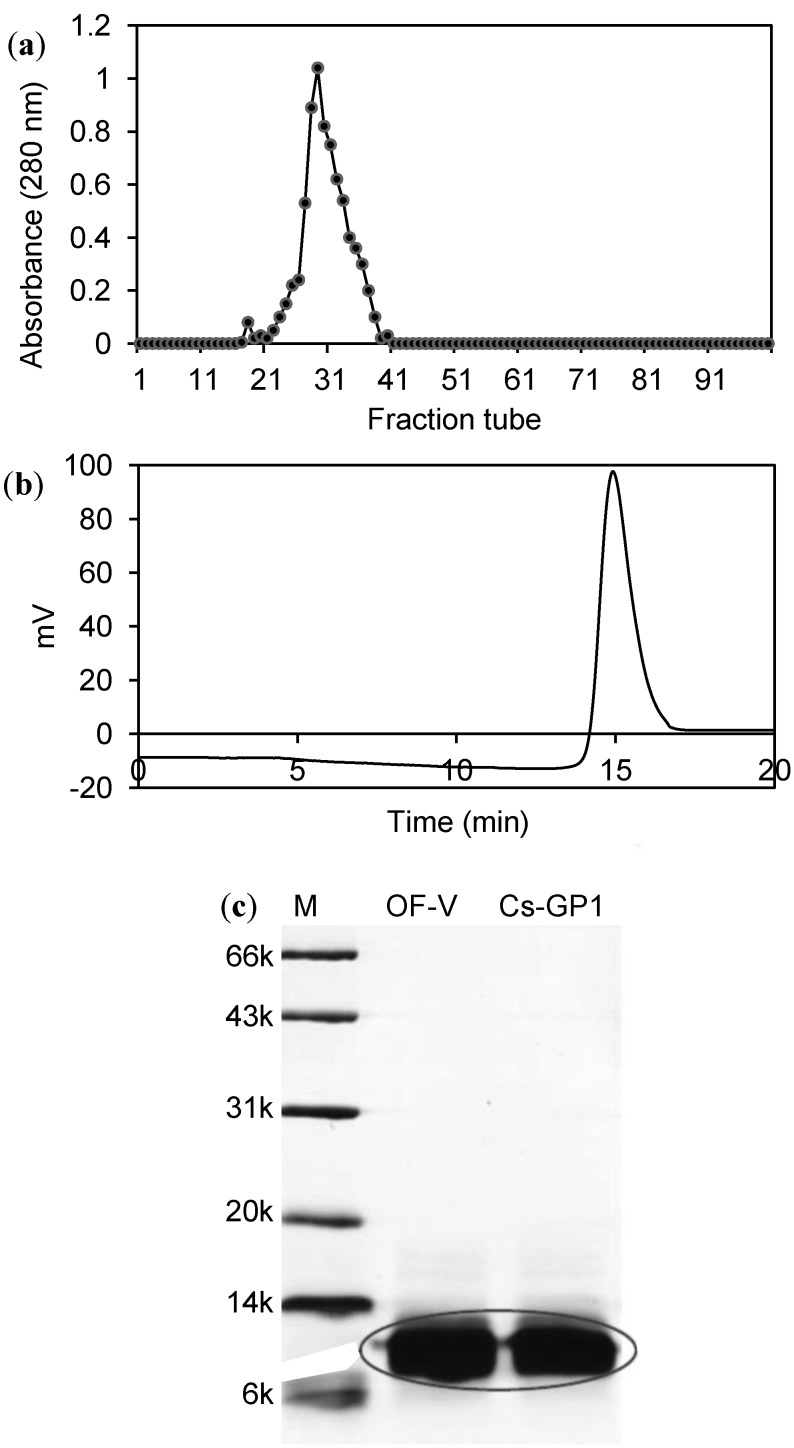
Fractionation and detection of glycopeptide Cs-GP1: (**a**) Ion exchange chromatograph purification of Cs-GP1 fraction on a DEAE 52 column (UV detection); (**b**) HPGPC profile of Cs-GP1 detected by on an Ultrahydrogel 250 column; (**c**) SDS-PAGE.

EPS-2 was fractionated into five fractions (OF-I, II, III, IV and V) by gel filtration through the Superdex 75 column with RI detection. OF-IV and OF-V had much higher protein contents (30%–50%) than the other three fractions (6% or lower) [12]. Fraction OF-V as well as fraction OF-IV (but not OF-I, II and III) also showed the protein absorption peak on UV at 280 nm. Because of its remarkable antioxidant activity, OF-V was further purified by ion exchange chromatography on the DEAE column (Figure 1a), yielding the glycopeptide fraction Cs-GP1. Cs-GP1 had a protein content of 52.3% (Table 1) and a carbohydrate content of 30% (determined by phenol-sulfuric method, data not shown), and was recognized as a glycopeptide.

**Table 1:** Composition and molecular weight of EPS-2 fractions obtained by gel filtration chromatography.

Fraction	Protein Content (%)	*M*w (kDa)	Mole Ratio				
			Man	Glc	GlcA	Gal	GalN
OF-IV	30.1	13	--	0.1	--	--	2.1
OF-V	50.5	6.0	1.0	3.2	1.5	1.0	0.96
Cs-GP1	52.5	6.0	1.0	3.2	--	0.2	0.3

“--”: Undetectable.

The Cs-GP1 fraction showed a single peak on HPGPC (Figure 1b), which was calibrated to an average *M*w of 6.0 kDa (Table 1). It exhibited a single thick band on SDS-PAGE (Figure 1c), corresponding to 8.0 kDa *M*w. The MALDI-TOF-MS spectrum of Cs-GP1 (Supplemental data Figure 1) revealed a major peak at 6057 *m*/*z* and two small fragments at 4423 and 1634 *m*/*z*, which were probably derived from the hydrolysis of the fragment at 6057 *m*/*z* during ionization. The fragment with *m*/*z* at 1634 was most likely the glyco-chain, whereas the *m*/*z* at 4423 was the peptide chain. These analytical results all indicated the molecular homogeneity of Cs-GP1, suitable for further structure analysis.

### 2.2. Sugar and Amino Acid Constituents of Cs-GP1

Monosaccharide analysis indicated that Cs-GP1 was composed mainly of glucose (Glc) and mannose (Man) at 3:1 mole ratio and a small proportion of GalN and Gal (Table 1) (Supplemental data: Figure 2 HPLC profile of Cs-GP1). Furthermore, the high contents of Glc and Man are indicative of an O-linked glyco-chain. As shown by the amino acid analysis (Table 2), Cs-GP1 had high mass contents of glutamic acid (Glu), aspartic acid (Asp), glycine (Gly) and cysteine (Cys) (76.6–40.6 μg/mg), but relatively low contents of threonine (Thr), tyrosine (Tyr) and histidine (His).

**Figure 2:**
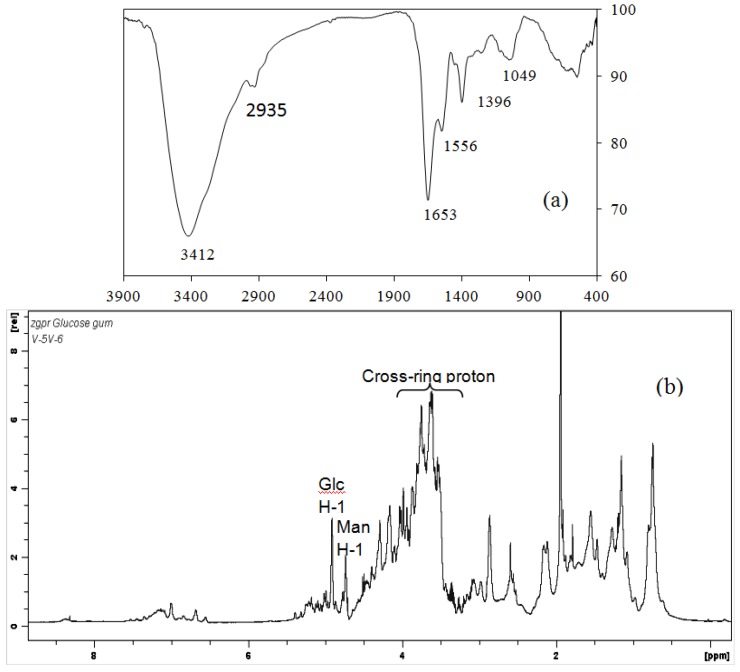
IR and NMR spectra of Cs-GP1: (**a**) IR; (**b**) ^1^H NMR.

**Table 2:** Amino acid composition of Cs-GP1.

Amino Acid		Content (μg/mg) *	Mole Ratio **	*M*_W_
1	Asp	66.89 ± 1.23	3.28	133
2	Thr	9.76 ± 0.31	0.54	119
3	Ser	33.56 ± 1.11	2.09	105
4	Glu	76.61 ± 2.41	3.40	147
5	Gly	40.61 ± 1.48	3.53	75
6	Ala	26.27 ± 0.93	1.93	89
7	Cys	40.61 ± 1.56	1.10	240
8	Val	7.86 ± 0.36	0.44	149
9	Ile	5.91 ± 0.44	0.29	131
10	Leu	7.12 ± 0.28	0.35	131
11	Tyr	3.96 ± 0.19	0.14	181
12	Phe	30.52 ± 1.13	1.20	165
13	Lys	30.22 ± 0.79	1.35	146
14	His	11.42 ± 0.52	0.48	155
15	Arg	26.61 ± 0.72	1.00	174
16	Pro	19.59 ± 0.67	1.11	115
Total amino acid		437.52 ± 16.15		

* Mean ± standard deviation (SD) of triplicate measurements; ** Mole ratio relative to Arg.

### 2.3. IR and NMR Spectra

Figure 2a shows the FT-IR spectrum of Cs-GP1. The peaks at 3407 and 2935 cm^−1^ are characteristic of O–H and C–H stretching vibration from the glyco-chain and amino acid, respectively. Between 1800 and 400 cm^−1^ are the characteristic bands of amino acids, *i.e.*, the peak at 1650.9 cm^−1^ assigned to amide I band from the peptide, the peak at 1560.7 cm^−1^ to amide group II vibration [13], and the peak at 1396.0 cm^−1^ to high content of COO–, which was probably from Asp and Glu [14]. The peak at 1054 cm^−1^ is attributed to C–O–C stretching vibration, and 846 cm^−1^ to α conformation in the sugar units. The molecular structure and composition deduced from IR are consistent with the results of monosaccharide and amino acid analysis (Table 1 and Table 2).

As for the ^1^H NMR spectrum (Figure 2b), the peaks between 8.1 and 8.5 ppm are assigned as reported previously [15,16] to the β-NH signals of amino acid, those between 6.5 and 7.5 ppm are assigned to α-NH signals, and between 4.8 and 5.5 ppm assigned to the anomeric signals of the sugar units. These peaks between 3.5 and 4.7 ppm are assigned to the C–H signals of both amino acids and sugar units, and those between 1.8 and 2.5 and 0.5 and 1.8 assigned to γ and λ C–H signals of the amino acid. The relatively strong signal between 4.0 and 4.6 and 3.6 and 3.9 may be attributed to the high contents of Gly, Ala and Asp; the peaks between 2.0 and 2.5 may be attributed to the H signals in Glu and Asp. These results are consistent with the above amino acid composition (Table 2).

### 2.4. Amino Acid Sequence of Peptide Chain

**Figure 3:**
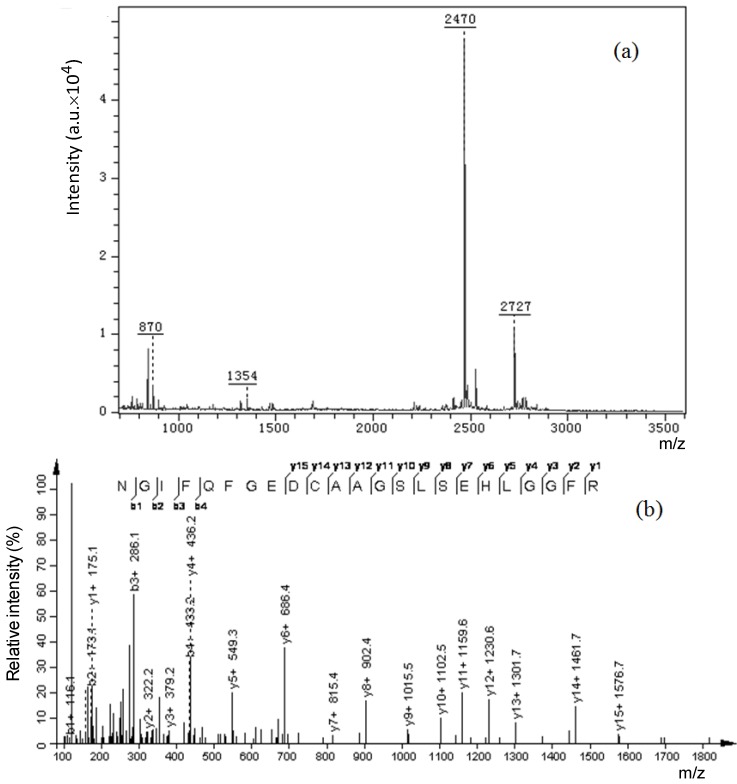
A peptide sequence from trypsin-digested Cs-GP1 detected by MALDI-TOF-MS/MS: (**a**) Spectrum of the peptide sequence; (**b**) Spectrum of its fragment with *m*/*z* at 2470.

After extensive in-gel trypsin digestion, Cs-GP1 was degraded into peptide fragments with *m*/*z* values ranging from 700–2800 (Figure 3a). Table 3 shows the *de novo* sequences of the peptide fragments detected by LC-ESI-MS-MS. The overall peptide sequence was derived from the overlapped sequences among the fragments as follows, GKNGIFQFGEDCAAGSLSEHLGGFREFREFLKAGNLE. The total mass (4102) was 321 *m*/*z* lower than the mass (4423) of the native Cs-GP1 derived from MALDI-TOF-MS, which was probably attributed to a small amount of peptide residual retained in the glyco-chain after enzymatic digestion. Based on the results from the sequence analysis and the above monosaccharide analysis, we suggest that the glyco-chain was attached to serine (Ser) in the peptide chain by O-linkage. The peptide chain sequence contained relatively large number of Ala (3), Gly (6) and Glu (5), which was consistent with the high contents found from amino acid composition analysis.

**Table 3:** *De novo* peptide sequences detected by ESI-MS/MS of trypsin digestion products of Cs-GP1.

Fragments	Mass	Sequence
A	1758	DCAAGSLSEHLGGFRE *
B	1412	AAGSLSEHLGGFR
C	1359	AGSLSEHLGGFR
D	2470	NGIFQFGEDCAAGSLSEHLGGFR
E	1930	KNGIFQFGEDCAAGSLSE
F	1987	GKNGIFQFGEDCAAGSLSE
G	2172	HLGGFREFLKAGNLE
Whole chain	4102	GKNGIFQFGEDCAAGSLSEHLGGFREFREFLKAGNLE

* Detected by both MALDI-TOF/MS/MS and LC-MS-MS (^n^ES).

The peptide chain sequence was further confirmed by MALDI-TOF-MS-MS analysis of the main peptide fragment with *m*/*z* at 2470 (Figure 3b). The fragment sequence was identified as NGIFQFGEDCAAGSLSEHLGGFR, which matched closely with the peptide chain sequence derived from LC-ES-MS-MS.

### 2.5. Antioxidant Activities

**Figure 4:**
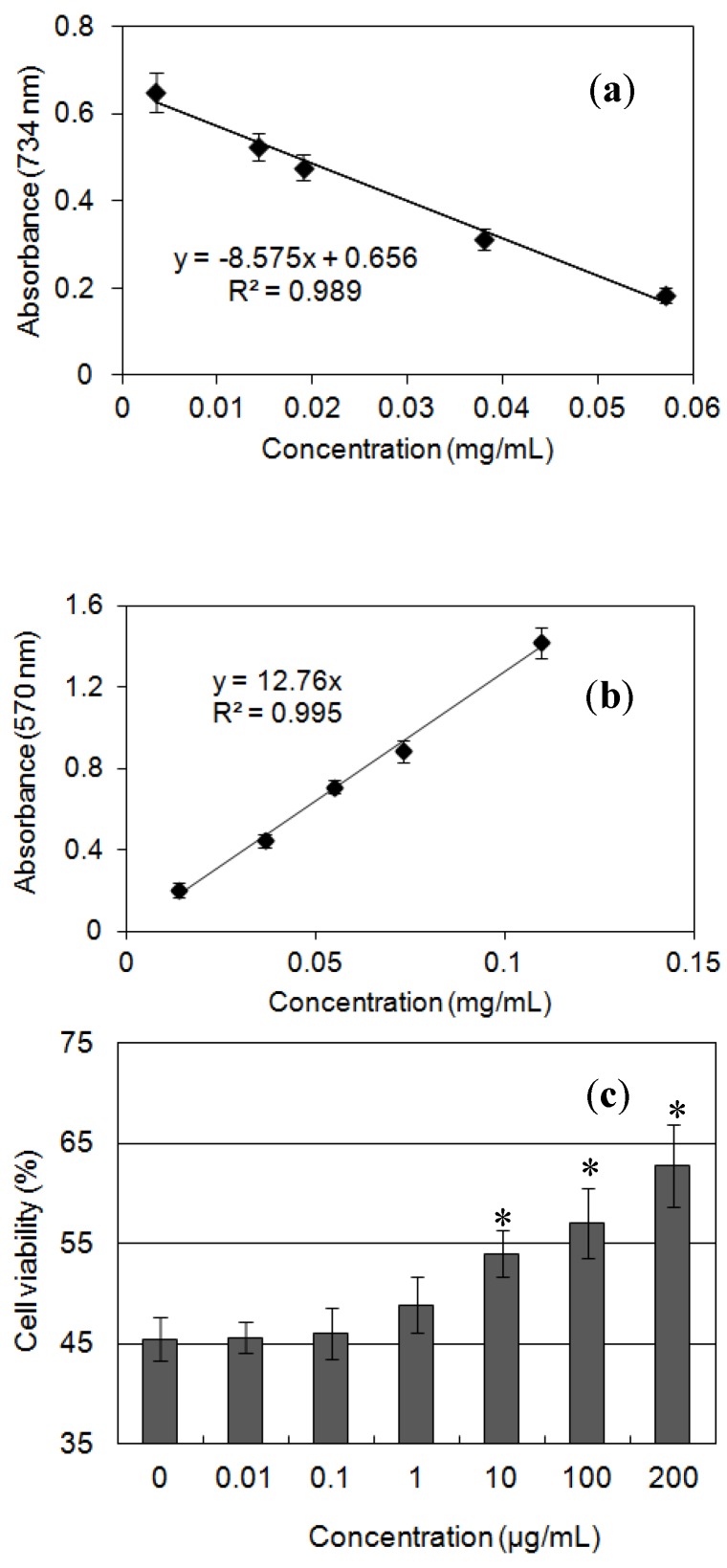
Antioxidant activities of Cs-GP1: (**a**) Scavenging (eliminating) ABTS^•+^ radicals; (**b**) Ferric reducing power (FRAP); (**c**) Protecting against H_2_O_2_-inducd viability loss of PC12 cells (exposed to 80 μM H_2_O_2_ for 1 h; * significant effect at *p* < 0.05).

Figure 4a shows the scavenging (or inhibiting) effect on ABTS^•+^ radicals and Figure 4b the ferric reducing power of Cs-GP1, both exhibiting a linear correlation with concentration. From these activity *versus* concentration curves, the following activity indexes were derived: IC_50_ of 35 μg/mL for inhibition of ABTS^•+^ radicals, TEAC value of 1180 μmol Trolox/g, and FRAP value of 610 μmol Fe (II)/g. The activity indexes for fraction IV derived from these two assays were IC_50_ 0.19 μg/mL on ABTS^•+^ radicals, 360 μmol Trolox/g and 43 μmol Fe (II)/g. In comparing these antioxidant activity indexes, Cs-GP1 had a much higher antioxidant capacity than OF-IV and the other three fractions (OF-I,II,III) which were composed mainly of carbohydrate as reported previously [12]. The strong antioxidant capacity of Cs-GP1 was also confirmed by the cell culture test (Figure 4c), showing a dose-dependent protecting effect against H_2_O_2_-induced cell viability loss of PC12 cells. The protective effect was statistically significant at *p* < 0.05 in the concentration range of 10–200 μg/mL and at 200 μg/mL maintained a cell viability of 63% after exposure to H_2_O_2_.

### 2.6. Discussion

Glycosylation is one of the most common posttranslational modifications of proteins in eukaryotic organisms and has significant influence on protein folding and intracellular trafficking [17,18]. The oligosaccharide moieties of glycoproteins are covalently bonded to the proteins in N- or O-linked form. Glycoproteins are involved in many important cellular communication processes associated with cell adhesion, host–pathogen interaction, and immune responses [19,20,21,22,23]. Therefore, the isolation and characterization of homogeneous glycoproteins and glycopeptides are needed for investigation of the biological functions and the structure–activity relationships.

Cs-GP1 isolated and fractionated from the low *M*w EPS of Cs-HK1 fungus has been identified as an O-linked glycopeptide with relatively high contents of Glc and Man in the oligosaccharide portion and its peptide portion contained high contents of Ala, Gly and Asp amino acids. Cs-GP1 showed strong antioxidant activity in both chemical and cell culture assays. There is ample literature on the strong antioxidant properties of naturally-occurring peptides or produced by hydrolysis of food proteins from plants and animals [3]. Most of the antioxidant food peptides are in the *M*w range of 500–1800 Da. The bioactivities as well as the properties of peptides are dependent on the amino acid composition and sequence. As free amino acids are not active in general, the amino acid sequences are crucial for the antioxidative activity of peptides. Some previous studies have suggested that high contents of some amino acid species such as Asp, Gly and Ala were significant factors for several antioxidative peptides from soybeans [24] and jumbo squids [25]. However, no general rule of thumbs has been established for the active amino acid composition and sequences.

## 3. Experimental Section

### 3.1. Materials

The Cs-HK1 fungus used in this study was previously isolated in our lab from the fruiting body of a wild *C. sinensis* organism [10] and has been preserved at the China General Microbiological Culture Collection Center (Registration No. 6004). Ultrahydrogel 250 columns were acquired from Waters Corp. (Milford, MA), DEAE-cellulose anion-exchange resin from Whatman (Brentford, UK), papain and cysteine from Fluka (Seelze, Germany). The protein *M*w markers of 6–66 kDa for SDS-PAGE were from GE Healthcare. The carbohydrate standards, d-mannose, l-fucose, l-arabinose, d-galacturonic acid and lactose were from Sigma (St. Louis, MO, USA), the derivatization reagent, 1-phenyl-3-methyl-5-pyrazolone (PMP) from Sinopharm Chemical Reagent Co., Ltd. (Shanghai, China), and the D_2_O (99.8%) for NMR from Cambridge Isotope Laboratories Inc. (Andover, MA, USA). Sequencing grade modified trypsin was from Promega (Madison, WI, USA), alpha-cyano-4-hydroxy-cinnamic acid (CHCA) and trifluoroacetic acid (TFA) were from Sigma, and formic acid (FA) and ACN from Merck (Darmstadt, Germany).

Trolox ([(*S*)-(2)-6-hydroxy-2,5,7,8-tetramethyl-chroman-2-carboxylic acid]) and 2,2'-azinobis(3-ethylbenzothiazoline-6-sulfonic acid) (ABTS) for the antioxidant assays were purchased from Calbiochem/EMD (Gibbstown, NJ, USA). The PC12 cell line for the cell culture tests was obtained from American Type Culture Collection, the RPMI 1640 medium, and fetal bovine serum from Gibco-BRL (Grand Island, NY, USA), H_2_O_2_ and MTT (3-(4,5-dimethylthiazol-2-yl)-2,5-diphenyltetrazolium bromide) from Sigma.

### 3.2. Isolation and Purification of EPS from Cs-HK1 Mycelial Culture

**Figure 5:**
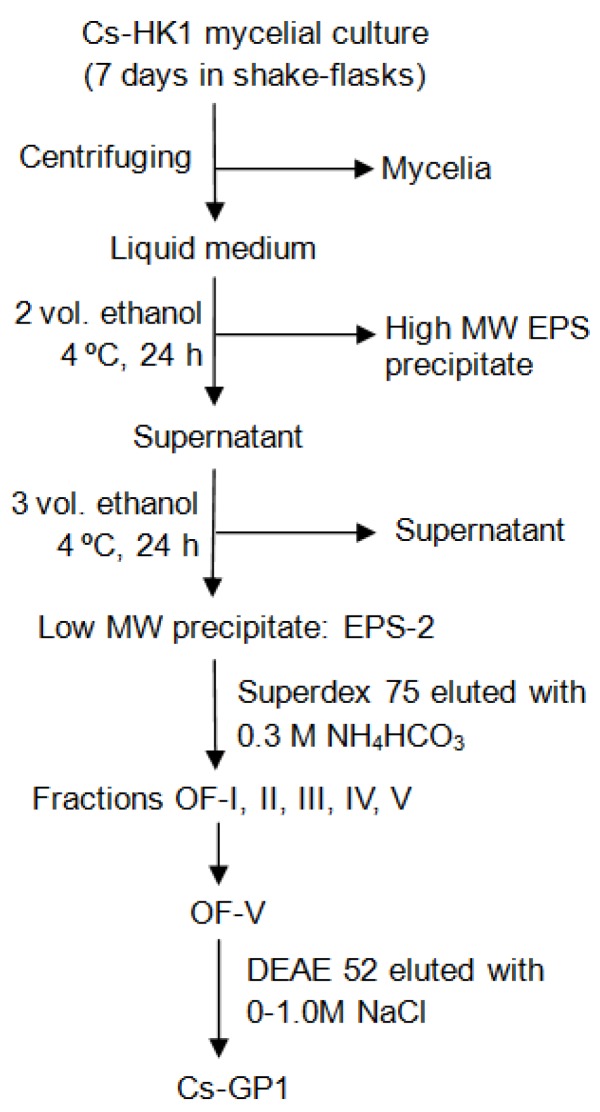
Procedure for isolation and fractionation of EPS from Cs-HK1 mycelium liquid medium and the purification of glycopeptide Cs-GP1.

Figure 5 illustrates the procedure for isolation and fractionation of EPS from the Cs-HK1 mycelial culture, and purification of the glycoprotein. The Cs-HK1 fungus was cultivated in 250 mL Erlenmeyer flasks each containing 50 mL of a liquid medium, shaken constantly at 150 rpm and 20 °C for 7 days [10]. The mycelial broth was then centrifuged and the supernatant liquid medium was collected for isolation of EPS by ethanol precipitation. The ethanol precipitation was performed in two steps, using 2-volume ratio of ethanol (96% grade) to the liquid medium in the first step to precipitate the high-*M*w EPS, followed by another 3-volumes of ethanol in the second step to precipitate the remaining low-*M*w EPS. The precipitate was washed with acetone, redissolved in water and lyophilized. EPS-2 (~0.3 g) was redissolved in 2 mL distilled water and loaded onto a Superdex 75 gel filtration column (2.6 × 60 cm), eluted with 0.3 M NH_4_HCO_3_ at a flow rate of 0.3 mL/min, and monitored by RI. The peak fractions collected were scanned by UV and the fraction (OF-V) exhibiting an absorption peak was collected as the glycopeptide fraction for the following experiments.

The OF-V fraction was dialyzed against distilled water and lyophilized. It was (~100 mg) then fractionated by anion-exchange chromatography on a DEAE-cellulose column (2.6 × 40 cm), eluted with NaCl on a linear gradient from 0 to 1.0 M (in 0.1 M sodium acetate solution at pH 5.0) for 500 min at 1.0 mL/min, and monitored with UV at 280 nm. The peak fraction was collected and dialyzed against distilled water and lyophilized, yielding the final glycopeptide Cs-GP1.

### 3.3. Analysis of Cs-GP1 Molecular Composition and Properties

#### 3.3.1. Monosaccharide, Amino Acid and Protein Contents

Monosaccharide composition was analyzed by HPLC as described by Chen *et al.* [16]. In brief, Cs-GP1 (~2 mg) was hydrolyzed with 2 M TFA at 110 °C in nitrogen atmosphere for 8 h, with lactose added as an internal standard. The hydrolysate was dried under vacuum, and then derivatized with 450 μL 1-phenyl-3-methyl-5-pyrazolone (PMP) solution (0.5 M, in methanol) and 450 μL of 0.3 M NaOH at 70 °C for 30 min. The reaction was stopped by neutralization with 450 μL of 0.3 M HCl, followed with chloroform extraction (1 mL, three times). The extract solution was analyzed by HPLC on a Waters 2870 instrument with an Agilent ZORBAX Eclipse XDB-C18 column (5 μm, 4.6 × 150 mm) at 25 °C with UV detection at 250 nm. The mobile phase was composed of 0.05 M KH_2_PO_4_ (pH 6.9) with 15% acetonitrile (solvent A) and 40% acetonitrile (solvent B) in water on a gradient from 8%–19% B in 25 min.

The amino acid composition was analyzed after hydrolysis of Cs-GP1 (with 6 M HCl under reduced pressure at 110 °C for 24 h) using a Hitachi 835-50 Amino Acid Analyzer (Hitachi, Tokyo, Japan). The protein content was determined by Lowry method using bovine serum albumin as a standard [26].

#### 3.3.2. Average Molecular Weight

The average *M*w of Cs-GP1 was analyzed by high pressure gel permeation chromatography (HPGPC) on a Waters instruments (Waters 1515 isocratic pump +2414 refractive index (RI) detector) and calibrated with dextran *M*w standards, as reported previously [11,12]. The *M*w distribution was also detected by SDS-PAGE (using 4% stacking gel and 12% separating gel) and staining with Coomassie Brilliant Blue in comparison with those of protein *M*w markers of 6–66 kDa. The Cs-GP1 sample was dissolved at 1 mg/mL in distilled water and added at 1:3 volume ratio into a buffer solution of 0.5% SDS with 1%-mercaptoethanol, and then heated to boiling for 5 min. The gels were stained with Coomassie Brilliant Blue R-250 to visualize proteins.

The molecular weight was analyzed more accurately by the matrix-assisted laser desorption ionization time-of-flight mass spectrometry (MALDI-TOF MS) on an ultrafleXtreme (Brukers, Germany), using CHCA as matrix. The Cs-GP1 sample solution was mixed with 1 volume of matrix solution (20 mg/mL of CHCA in acetonitrile/water, 50:50, *v*/*v*) in a final concentration of 50 μg/mL. Finally, 0.5 μL of the mixture was deposited onto the MALDI target plate. All spectra were the results of signal averaging of 200 shots. The instrument was operated at laser energy 20% (coarse) and 60% (fine), and resolution 1000.

#### 3.3.3. NMR and IR Spectroscopy

NMR and IR spectroscopy were performed with the same procedures and instruments as described previously [12,15]. In brief, ^1^H NMR was performed at 600 MHz and ^13^C NMR at 150 MHz at room temperature on a Bruker AVANCE III 600 spectrometer with Topspin 3.0 software for data processing. The Cs-GP1 sample (~30 mg) was lyophilized with 500 μL D2O (99.8%) twice and then dissolved in 500 μL high quality D2O (99.96%) containing 0.1 μL acetone as an internal standard for the ^1^H chemical shifts. Infrared (IR) spectrum was recorded on a Perkin-Elmer 1600 instrument at room temperature in wave number range of 4000–400 cm^−^^1^.

### 3.4. Analysis of Peptide Chain Sequence

#### 3.4.1. In-Gel Digestion

SDS-PAGE was performed as described in Section 3.3.2. The gel was washed with Milli-Q water and then stained with 50 mL of Coomassie staining solution containing 45% (*v*/*v*) methanol, 10% (*v*/*v*) acetic acid, and 0.15% (*w*/*v*) Coomassie Brilliant Blue R350 for 1 h, then finally de-stained for 1–1.5 h using 100 mL of destaining solution containing 40% (*v*/*v*) methanol and 10% (*v*/*v*) acetic acid in Milli-Q water. The gel stained by Coomassie was sliced into four pieces of gel matrix, and then cut into about 1 mm cubes using a razor blade on a clean glass surface. The cubes were transferred into Eppendorf tubes for in-gel trypsin digestion. The in-gel trypsin digestion and the extraction of the tryptic fragments were performed according to well-established protocols [27]. In brief, acetonitrile (ACN) (25–35 μL) was added to each tube to dehydrate and shrink the gel pieces. After drying with Speed-VAc, the gel pieces were incubated in 25–35 μL of digestion buffer (0.1 μg/μL sequencing grade modified trypsin in 50 mM NH_4_HCO_3_) for 45 min in an ice water bath. The mixture was centrifuged at 10,000× *g* and the supernatant extract was collected. The remaining gel pieces were incubated in 20 μL of 20 mM NH_4_HCO_3_ for 10 min and the supernatant was collected and combined with previous extract. The remaining gel pieces were extracted with 20 μL extraction buffer (50% ACN, 5% formic acid) for 20 min and the extract was combined with the previous ones. Finally, the combined extract was dried by Speed-Vac and stored at −80 °C until MS analysis. The tryptic digestion product of Cs-GP1 was subject to MALDI-TOF analysis as described in Section 3.3.2 to monitor the degree of degradation and then applied to LC-MS/MS and MALDI-TOF-MS-MS for analysis of the peptide sequences.

#### 3.4.2. Mass Spectrometry

For the LC-MS/MS analysis, the digested product samples were desalted using the ZipTip-C18 (Millipore) treatment. The samples were then loaded onto an analytical column (15 cm × 75 μm i.d.; Acclaim@PepMap100 C18, Dionex, Sunnyvale, CA, USA). The nano-flow was eluted at a flow rate of 300 nL/min with solvent A (2% ACN with 0.1% formic acid) and solvent B (95% ACN with 0.1% formic acid). LC analysis was performed on a 40 min staged gradient elution program, 0–4 min (5% B), 4–40 min (5%–35% B). The column outlet was coupled directly to a high voltage ESI source, which was interfaced to a Shimadzu UFLC-LTQ-Orbitrap HCD, operated at 1.7 kV spray voltage in the nES-LC-MS/MS mode in 200–2500 Da *m*/*z* range.

The peptide *de novo* sequences were derived by matching the acquired data with the National Center for Biotechnology Information (NCBI) non-redundant protein database (fungi) using the MASCOT software package (Version 2.3, Matrix Science, London, UK). The peptide mass and MS/MS tolerance were both 0.2 Da. The peptides have the allowance of one tryptic missed cleavage, one fixed modification with carb-amidomethyl (C) and one variable modification by oxidation.

MALDI-TOF-MS-MS analysis was performed as described in Section 3.3.2. The trypsin digested peptide was subjected to MS/MS analysis and the results were used to confirm the data from LC-ES-MS-MS.

### 3.5. Antioxidant Activity Assays

The antioxidant activities of Cs-GP1 as well as other EPS fractions attained from were evaluated using two chemical assays, the Trolox equivalent antioxidant capacity (TEAC) and the ferric reducing ability of plasma (FRAP) assay, and a cyto-protection test using H_2_O_2_-induced cell injury, as described previously [11]. In brief, the TEAC assay measures the ability of a compound to eliminate or scavenge ABTS^•+^ radicals using Trolox as a response reference [28]. The EPS sample solution in water was reacted with the ABTS^•+^ solution for 20 min at room temperature, followed by measurement of the absorbance at 734 nm. The scavenging activity of a sample was correlated with the absorbance decrease, and converted to a TEAC value in μmol Trolox/g sample by calibration with Trolex from 0–30 μM. The FRAP assay was performed according to Benzie and Strain [29]. The FRAP reagent was reacted with the EPS sample for 15 min at room temperature, followed by measurement of absorbance at 593 nm. The reducing power of a sample was correlated to the absorbance increase, and converted to a FRAP activity (μmol Fe(II)/g sample) by calibration with ferrous sulfate from 0–30 μM.

The cyto-protective activity of EPS fractions against oxidative cell damage was tested in rat pheochromocytoma PC12 cell culture, subjected to peroxide H_2_O_2_ treatment [11,12]. The EPS samples were pre-dissolved in phosphate buffered saline (PBS) at 10 mg/L. The PC12 cell culture was maintained in RPMI 1640 medium supplemented with 10% fetal bovine serum in a CO_2_ incubator at 37 °C. The activity test was performed on a 96 well-plate by treating the cells with 80 µM H_2_O_2_ and EPS sample solution at selected concentrations (0.001–200 μg/mL). The cell viability was measured by the MTT assay and represented in percentage relative to the native culture (N) to culture without any treatment.

## 4. Conclusions

An antioxidative glycopeptide Cs-GP1 has been isolated from the low *M*w fraction of EPS produced and released by the Cs-HK1 fungus into the liquid medium. Its molecular composition and structure have been partially characterized including the monosaccharide, amino acid composition, and the peptide chain sequence through hydrolysis and analytical experiments, though its glyco-chain structure remains unknown. Another distinct feature of Cs-GP1 is its presence as an extracellular product which is favorable for mass production by liquid fermentation and efficient recovery from the liquid medium. In other words, the present study has demonstrated the application of a medicinal fungus as the source or producer of novel bioactive glycopeptides.

## Supplementary Materials

Supplementary Figures can be found at http://www.mdpi.com/1422-0067/15/10/17318/s1.

## Supplementary Files

Supplementary File 1
