# Supplementary material for: Isolation and Structure Characterization of an Antioxidative Glycopeptide from Mycelial Culture Broth of a Medicinal Fungus
Source: Int J Mol Sci. 2014 Sep 29;15(10):17318–32. doi: 10.3390/ijms151017318 (PMC4227164; doi:10.3390/ijms151017318)
Supplement: Supplementary File 1 [file ijms-15-17318-s001.pdf]

Supplementary Information

Figure 1. MALDI-TOF-MS spectra of Cs-GP1.

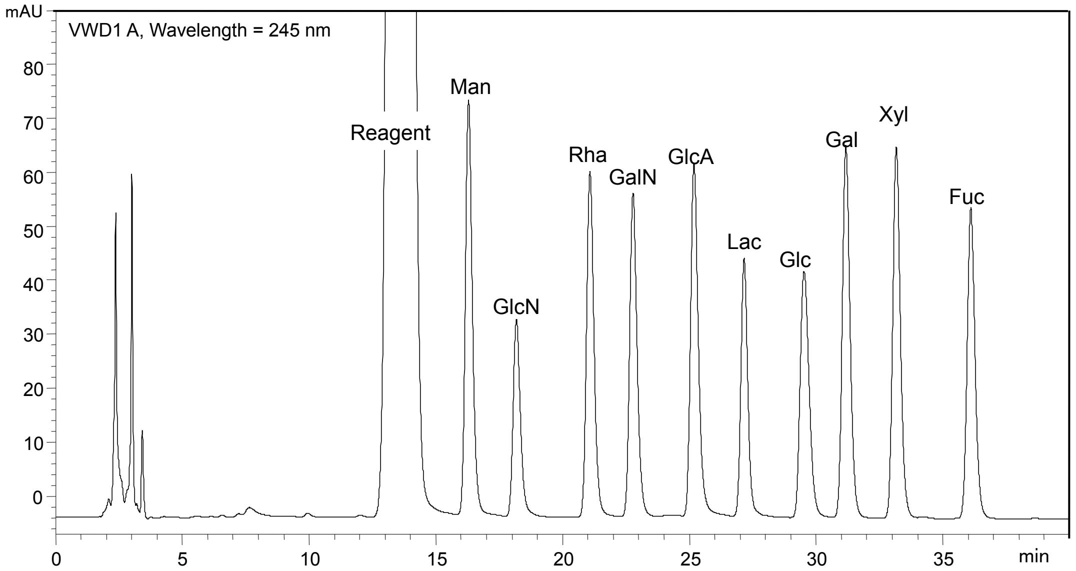

Figure 2. Monosaccharide composition of Cs-GP1 by HPLC: (**a**) monosaccharide standards; (**b**) glycoprotein Cs-GP1.

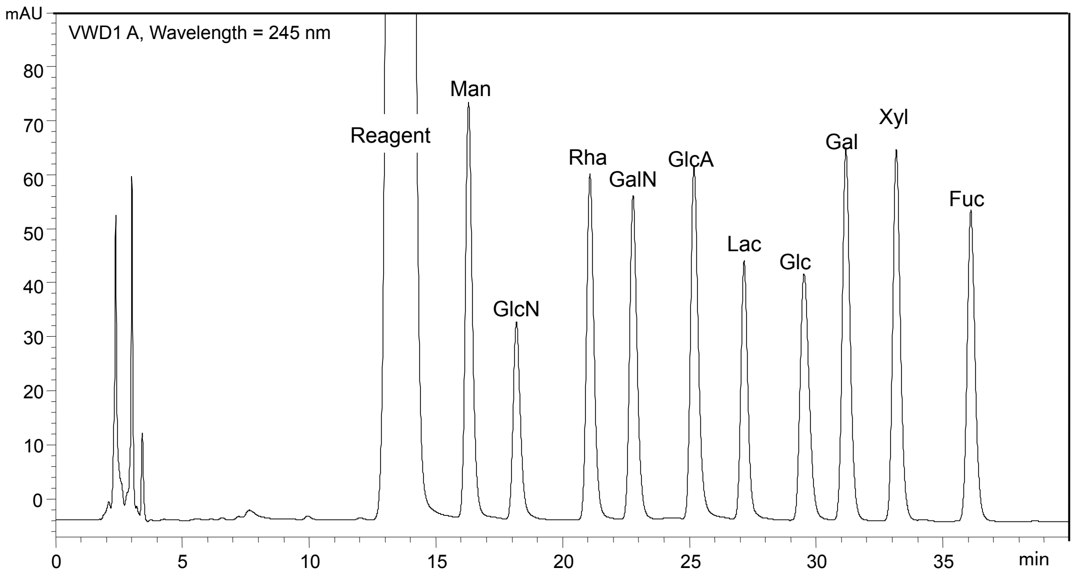

(**a**)

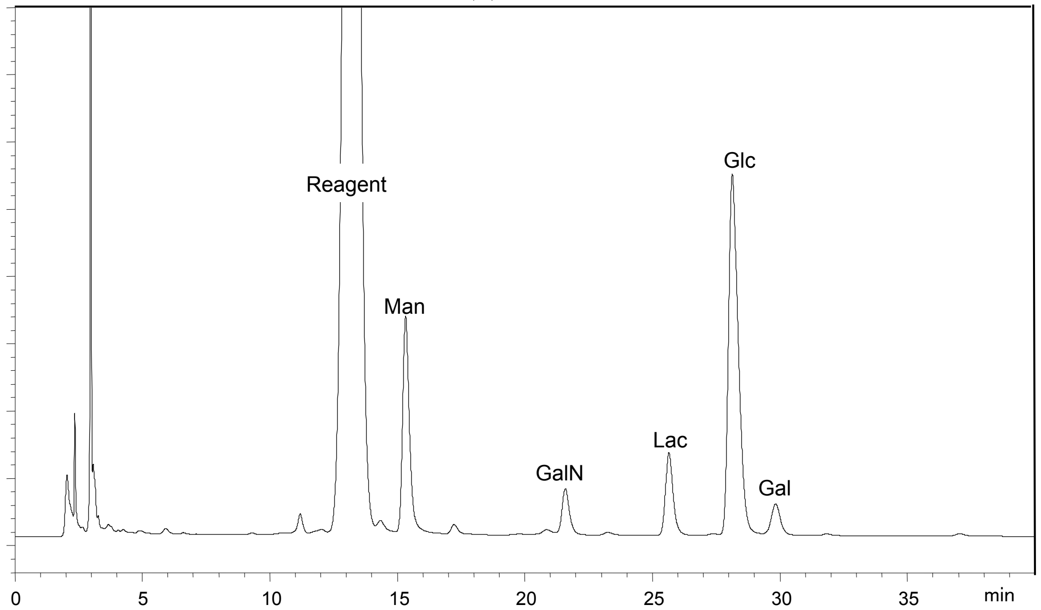

(**b**)
